# Developing Leadership Among Dental Residents: An Exploratory Study

**DOI:** 10.7759/cureus.36600

**Published:** 2023-03-23

**Authors:** Hawazen Radwan, Sami Al-Nasser, Abdullah Alzahem

**Affiliations:** 1 Department of Dental, Prince Sultan Military Medical City, Riyadh, SAU; 2 Department of Medical Education, King Saud Bin Abdulaziz University for Health Sciences College of Medicine, Riyadh, SAU

**Keywords:** post grad medical education, health care leadership, dental professional, competence by design, residency program

## Abstract

Introduction: Today's healthcare requires leaders to lead and improve the healthcare sector. CanMEDS framework is the one defining the competencies required for all Saudi residency programs, including dental specialty. Senior residents should demonstrate readiness to transition to practice as a leader. It is imperative to understand the notion of medical education and its influence on the training of future dentists. The major significance of this study is exploring the current leadership development and the integrated training into the Saudi Board Dental Residency Program that has not been systematically studied.

Methods: This was a qualitative study employing the phenomenological approach. The theoretical saturation point determined the sample size using a purposeful sampling technique. Semi-structured interviews were used for data collection using a semi-structured interview guide. A descript platform was used for the recordings’ transcription. Ongoing thematic data analysis was done using Nvivo computer software by QSR International. Themes were generated, and the data were interpreted within supported with the most relevant quotations.

Results: Sixteen senior residents were required to serve the study purpose. Three themes emerged: awareness of leadership, educational experience, and factors that impacted leadership development. Awareness of residents about the leader's role was limited. Residents developed leadership under the training program with inconsistency and lack of structure. Summative reports were received as part of the assessment, whereas no integral protocol for formative feedback. Specialties, training centers, and coaching were identified as factors that impacted leadership development.

Conclusion: This study highlighted leadership development during the residency period. The residents struggled and varied in developing leadership skills relying on their educational experience and learning environment. Residency programs may verify equivalent “leader role” education for all specialties and training centers in residency training in Saudi Arabia. Dovetailing leadership coaching with daily teaching workflow and implementing faculty development initiatives to allow for appropriate feedback and assessment of these skills are advised.

## Introduction

The Saudi Commission for Health Specialties (SCFHS) adopted the CanMEDS framework to define the competencies for all residency programs, including dental specialty, under its purview [1]. The medical expert role is central to the CanMEDS framework and draws on the competencies, including the manager role [2]. In 2015, CanMEDS changed the Role from “Manager” to “Leader” which reflects an emphasis on the leadership skills needed as they recognized the increase in complexity of health care [3]. Residents develop competencies at different stages throughout their residency period and before practice displayed at Competence by Design (CBD). In the final stage, concerning the leader role, the senior trainee should demonstrate readiness to make the transition to autonomous practice as a leader. Board certification will be granted upon successfully completing the “transition to practice” stage [2,4]. The positive, collaborative frame of the CanMEDS 2015 Leader Role encourages physicians to develop and use leadership skills to advance the care of their patients as well as contribute to improving the healthcare system. The physician leader is an engaged individual who takes the initiative to contribute in a collaborative way and work towards positive and sustainable change in health care from the level of an individual patient to the level of the health care system [4]. That aligned with the Saudi vision of 2030 of the healthcare sector. Since it requires leaders who make it possible for the most capable and appropriate individuals to take charge of leading and improving health care in Saudi Arabia [3]. The development of leadership skills must become a standard part of medical training at all levels and in all specialties [5]. Leaders do not need a formal title to lead. Leaders also recognize that they do not necessarily need to be in charge [3]. Moreover, dentistry is currently undergoing profound changes in structure and policy nationally and internationally. Therefore, it is imperative to understand the notion of medical education and its overarching influence on the training of future dentists. Despite all this, research and development surrounding leadership outcomes in the dental profession are generally limited [6]. On the undergraduate level, one study was done at King Abdulaziz University (KAU), Saudi Arabia. The study aimed to conduct a contextual analysis of interviews intended to assist with the future design of a feasible and relevant leadership and management course for undergraduate medical students. The target group was healthcare leaders with no student involvement. Multiple core categories for a leadership and management curriculum emerged with many interrelated themes. Most participants mentioned that leadership could be taught and that early exposure is beneficial for developing skills [7]. In 2021-2022, an integrated national program called Mulhem was offered by Academic Affairs and the Healthcare Leadership Academy (HLA) at the SCFHS. The program is dedicated to supporting residents and fellows of the SCFHS programs, including dental, with the leadership skills and competencies necessary to lead multidisciplinary health teams with confidence and efficiency. Applicants go through a rigorous selection process to be able to enroll [8]. Nevertheless, information on how the leader role is incorporated in postgraduate dental education specifically is bounded. To meet this need, an exploratory in-depth qualitative study was suggested to evaluate the current leadership development and the integrated training into the Saudi Board Dental Residency Program. This information is vital for further research and improvement initiatives. To the best of our knowledge, no such study was conducted in Saudi Arabia. The findings of this study will contribute to advancing leadership and activating the “leader role” as competency in residency training.

## Materials and methods

This study was a qualitative study employing a phenomenological approach [9]. The Institute Research Board (IRB) approved the study protocol at King Abdullah International Medical Research Center (Reference number IRB/1909/22). The study was conducted in the dental center at Prince Sultan Military Medical City (PSMMC), Riyadh, Saudi Arabia. The SCFHS has recognized it as a dental residency training center for different dental specialties. Those include nine specialties: oral maxillofacial surgery, periodontics, endodontics, prosthodontics, pedodontics, orthodontics, restorative dentistry, family dentistry, and oral medicine. All residency programs of the above specialties are competency-based and follow the CanMEDS 2015 framework. They are all three years long, except oral maxillofacial surgery is five year-long, and oral medicine is four year-long. The first year (R1) and second year (R2) are junior levels, while the third (R3) and above are considered to be senior levels. In this study, we recruited senior-level residents who are ready to transition to practice.

The sample size was determined using the theoretical saturation point, at which additional interviews stopped providing new insights into the questions being explored. We utilized a purposeful sampling technique that enabled the researcher to interview the most valuable participants to address the aim of the study.

Data collection

**Figure 1:**
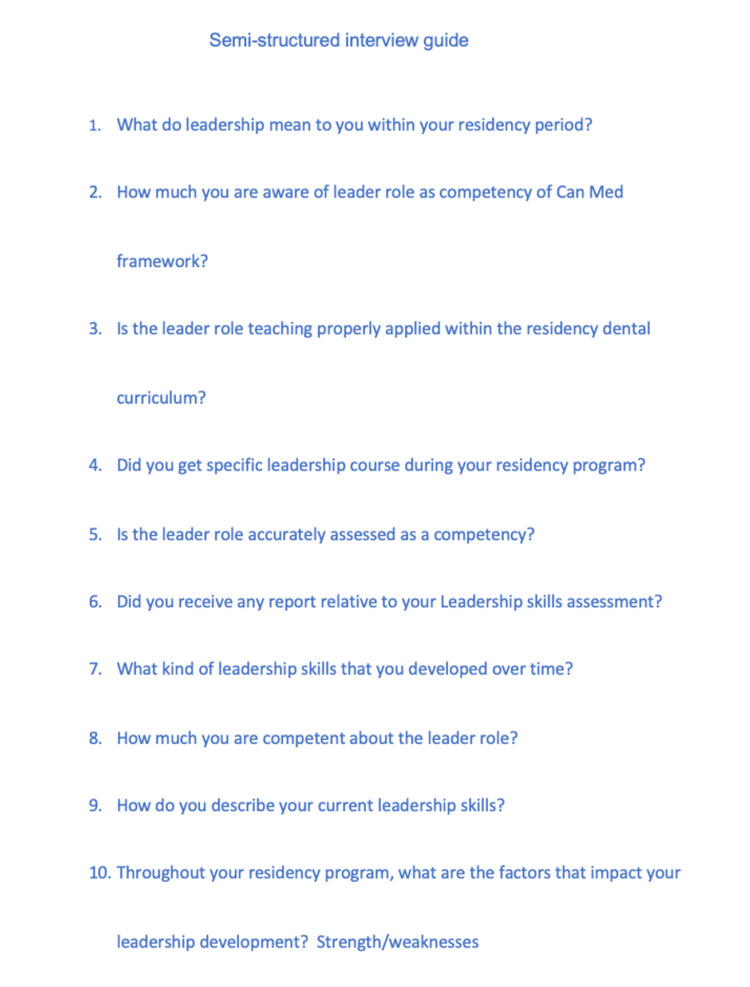
Interview guide: the questions that were used for the semi-structure interviews

A 1:1 semi-structured interview with the targeted residents and answering open-ended questions about their leadership development during their residency period were used for data collection. They were conducted with residents in English face-to-face and via Zoom using a semi-structured interview guide developed after reviewing the relevant literature and the CanMEDS leader role key competencies description. They were piloted on three participants. The interview questions order varied from interview to interview with possible added questions. The questions are presented in the interview guide (Figure 1). The interviewer/author was not familiar with the residents personally, just known by name in the dental center as a continuous quality improvement and patient safety representative. The selected participant was invited personally or by phone to contribute with an introduction to the study and a copy of the approved informed consent, which stated the agreement for participation to record and transcribe responses. Also, it stated that responses would be kept anonymous and used for the study purpose only. Once a resident accepted to participate, an interview was conducted between September 20 and November 20, 2022. Each interview took 30-45 minutes. No financial incentives or other were provided to the participants; however, gratitude and appreciation were delineated at the end of the interview.

Data analysis

**Figure 2:**
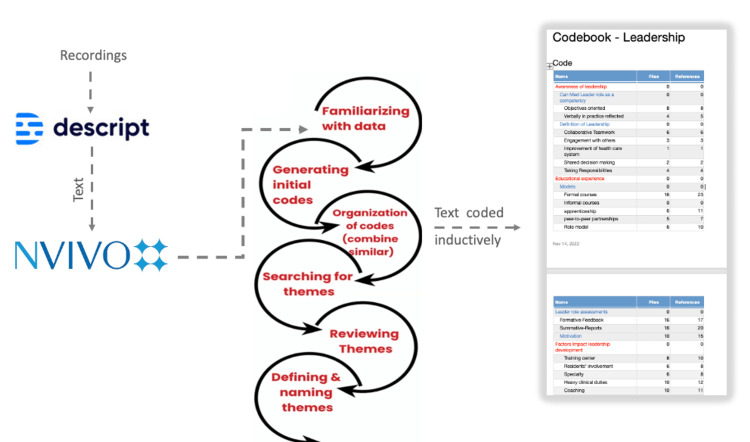
Data analysis including transcription, coding, and themes generation

A descript platform was used for the recordings’ transcription. Ongoing data analysis was done using Nvivo computer software by QSR International. The transcripts were read multiple times to familiarize with the data for thematic analysis, where the text was coded inductively given the most relevant and appropriate codes. Themes were generated by identifying patterns in the data and merging similar codes. The data were interpreted within the themes and supported with the most relevant quotations (Figure 2).

The study objectives were revisited regularly to keep them under focus while themes and concepts emerged. The corresponding author did all codings, which were reviewed and confirmed by the other two authors. Constant comparison and iteration were implemented to identify emerging themes in all stages of analysis. The themes review was done before interpretation. The research team used to have periodic online meetings to review and debate findings, followed by logical editing of code names. All the research team members agreed on the final findings. The themes were finalized, and the codebook was exported to a word document showing themes, subthemes, the number of files coded, and references (Table 1).

**Table 1:** The codebook showing themes, subthemes, number of files coded, and references

Name	Files	References
Awareness of leadership	0	0
CanMEDS Leader role as a competency	0	0
Objectives oriented	8	8
Verbally in practice reflected	4	5
Definition of leadership	0	0
Collaborative Teamwork	6	6
Engagement with others	3	3
Improvement of healthcare system	1	1
Shared decision-making	2	2
Taking responsibilities	4	4
Educational experience	0	0
Motivation	10	15
Models	0	0
Formal courses	16	23
Informal courses	0	0
Apprenticeship	6	11
Peer-to-peer partnerships	5	7
Role model	6	10
Leader role assessments	0	0
Formative-Feedback	16	17
Summative-Reports	16	20
Factors Impacted leadership development	0	0
Training center	8	10
Residents’ involvement	6	8
Specialty	6	8
Heavy clinical duties	10	12
Coaching	10	11

The thematic analysis steps prescribed by Braun and Clarke (2014) informed our analysis process and theme generation [10].

## Results

Demographic characteristics

Sixteen Saudi board dental senior residents (excluding the three residents in the pilot phase) were required to serve the study purpose. Five participants were acting as chief residents for their batch. Gender selection was even among participants (female: n=8 (50%), male: n=8 (50%)). Those include eight specialties as follows: oral maxillofacial, periodontics, endodontics, prosthodontics, pedodontics, orthodontics, restorative dentistry, and oral medicine.

Thematic analysis

**Figure 3:**
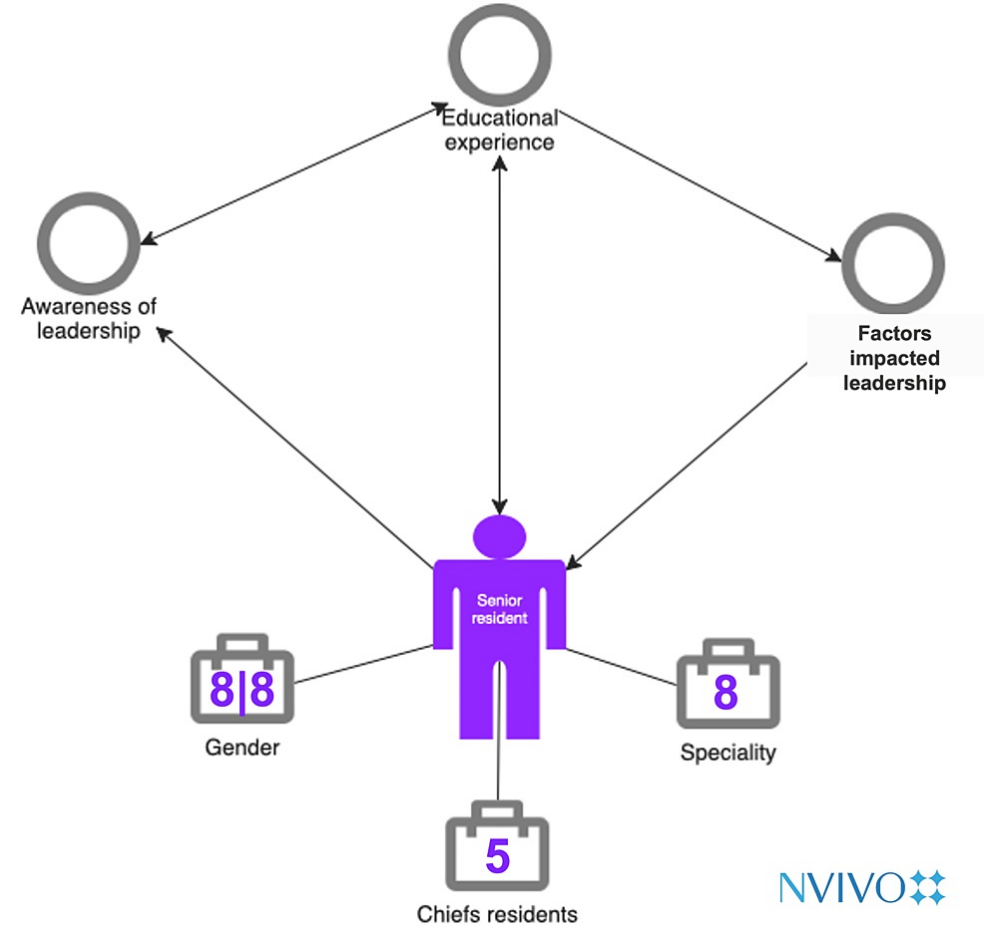
Concept map of thematic analysis using NVIVO computer software by QSR International

 Three main themes emerged as awareness of leadership, educational experience, and factors that impacted leadership development (Figure 3).

Awareness of leadership

Definition

Residents perceived the definition of leadership in five different categories. Those involved: Taking responsibility, collaborative teamwork, sharing decision-making, improving the healthcare system, and engagement with others. A higher number of coding was recorded under the collaborative teamwork category. A third-year male resident explained leadership: “It is a very complex term. I view it as someone who can lead a team toward a successful place. leadership is not about the person itself; it's about the collaborative teamwork”.

CanMEDS Competency

Residents struggled to identify some terms relative to the leader's role as stewardship and emotional intelligence. Residents were unclear about having them as an objective written or a concept verbally mentioned during the practice. A four-year female resident commented, “I’ve never seen it written or included maybe in the objectives during the orientation. However, I got the concept from day-to-day workload, day-to-day practicing within the department, seeing how seniors when I was a junior, and how consultants deal with seniors. Hence, it's mostly like an inherited thing”. Meanwhile, chief residents were more oriented toward the leader role enabling competencies. A third-year female chief resident commented, “I think I am aware enough about this competency as a part of my responsibility because I'm taking basically the leader of these settlements of competencies framework”.

Educational experiences

Motivation

Residents were substantially motivated toward leadership during their residency period as a tool for success, managing critical situations, and creating a healthy work environment. A third-year male resident mentioned, “At some point, you have actually to deal with things as a leader. Otherwise, you won’t succeed”. Another third-year female resident highlighted, "As a chief resident, I got that part of leadership on a personal level. On hard times, like the period of covid, I got to lead and take decisions”.

Educational Model

Residents listed various educational models relative to the leader role, but the structure was insubstantial within the program and inconsistent relative to the training centers and specialties. Seven out of 16 residents indicated that their residency programs provided formal leadership courses which are not routinely taught in other specialties. A third-year female resident mentioned, “We received a program called leadership, and it was online lectures along with the material and workshops. It was specific under the umbrella of our specialty”. However, four residents reported a lack of receiving any leadership model.

Other residents reported more informal leadership models. Four-year male resident highlighted apprenticeship and peer-to-peer partnership, “Working very closely with consultants and seniors teach us a lot of skills. When you're senior to just joining the junior, help them and show them how things can get done”. A four-year female resident acknowledged her supervisor as an undeniable role model, “Sometimes you will have the chance of working with a great example of leadership model, and I think subconsciously you will follow on to these”.

Assessment

The residents receive summative reports quarterly as part of the assessment; however, they did not recognize specific points of the leadership. A third-year female resident replied, “It is a part of the evaluation but nothing specific to leadership”. Moreover, there was no integral protocol for formative feedback. A four-year male resident explained, “Feedback is a genuine kind of guidance and advice more than official one”. Only two residents reported that “we receive it yearly for every rotation with feedback about communication and behavior”.

Factors impacted leadership development

Training Center

For many residents, the training center significantly impacted their leadership development. A fourth-year female resident replied, “Honestly, I would say that the medical city. I can see that training center is the influence but not the curriculum”. Another third-year male resident confirmed, “I found a difference from center to center; some centers provide more than just the base of the character”. They mentioned that being in a tertiary hospital setting, including a complex system, high patient flow, diversity of clinical cases, and a huge number of staff, gave them a chance to develop that leadership within the work environment. A fourth-year male resident added, “Training center relative to the high patient flow and the number of staff including the consultants and the auxiliary staff to deal with”. Moreover, they developed their commitment to safety culture, incident reporting, and patient safety as a part of the training center’s culture, policies, and procedures. A third-year male resident confirmed, “I got more exposure to patient safety and quality here”. It strengthened their leadership skills positively. On the other hand, two residents reported that the difference from center to center creates unhealthy competition between the training centers, negatively affecting the resident’s leadership development. A fourth-year male resident mentioned, “I can notice comparison and competition between the training centers that unhealthy”.

Specialty

Specialty is another factor that residents mentioned relative to leadership development. Specialties based on teamwork and peer partnership within the same specialty or interdisciplinary showed marked growth. A fourth-year male resident affirmed, “The specialty has a major role; working in a team improved my leadership skills even without asking”. A third-year female resident added, “As well interacting with other specialties like Interdisciplinary makes me more competent with leadership”. However, growth remains constant in specialties that do not necessitate teamwork and interactions. A third-year male resident mentioned, “Everyone is standing alone with no communication, working separately. We did not gain any growth out of the residency, and nothing was added to us”. Many residents believed that residents involvement reflected in their leadership skills. As they got involved in decision-making, responsibilities, and tasks led to progress in their ability to lead through experiences and exposure. A third-year male resident supported that, “Personal opinions of the resident when things happened, when discussions happened, I was given the opportunity or the freedom to decide. It allowed me to explore my opinions or actions that made me confident enough”. A third-year male resident elaborated, “We are responsible for our annual meeting where most of the residents interact with each other. We must prepare it at least two months ahead, organizing as well as preparing for scientific debates. We developed skills such as being efficient and willing to lead, which I think is important to acquire during residency. This is a crucial factor”. On the contrary, four residents reported the absence of any kind of involvement, which reflected as an area of stagnation. A third-year male resident stated, “It was not about leadership. I am, as a chief resident, just communicating the information to my residents”. Most residents struggle to develop leadership skills within heavy clinical duties as they focus on fulfilling the clinical requirements. A third-year male resident, “The amount of clinical work that you feel overwhelmed with the clinical context required, leaving the resident not interested enough. I mean just wants to get things done anyway”.

Coaching

Several residents highly acknowledged how supervisors provide coaching by drawing attention and reflecting upon their interactions besides their scientific knowledge. A third-year female resident affirmed, “Instructors and directors should not be in that position just because they are scientifically good. They should be willing to teach the resident to make them better leaders. I learned a lot of leadership skills guided by my director”. However, some program directors and supervisors are only oriented toward teaching science and clinical skills. Two residents reported that supervisors take the total lead in managing situations to have them focus on the clinical requirements only. A third-year male resident highlighted, “Supervisors are always around; we have them if anything happens. We didn't get any problem. I mean, even if we have, someone there would handle it normally; they take care and deal with everything”. Conversely, four residents mentioned that supervisors leave the quiet lead to the residents. A third-year female resident, I'm working myself relatively. The program is not oriented toward developing any leadership skills.

## Discussion

This study aimed to explore leadership development in Saudi dental residency programs at PSMMC in Riyadh, Saudi Arabia. The aim of the study was achieved through semi-structured individual interviews. Residents developed leadership as they progressed through their residency period. The implications of our study findings are significant.

First, senior residents offered only the general definition of leadership. They perceived a wide range of leadership meanings based on his/her individual experience, from taking responsibilities to maintaining and improving the entire healthcare system. This understanding is relevant to the definition of being a physician leader [2,3]. Most were toward collaborative teamwork, reflecting overlapping perceptions. This finding was consistent with the previous studies where residents overlapped leader roles with other roles such as communicator and professional [11]. Meanwhile, most residents’ responses were passive relative to the primary CanMEDS leader role key concepts like stewardship, emotional intelligence, and safety culture. Leader role key and enabling competencies recognition were limited only to chief residents. That contradicts what the Saudi board system states, which codifies an emphasis on the leader role in its CanMEDS framework [1,2]. The reporting by chief residents regarding their awareness was also observed in another recent study which is consistent with our findings. While all residents are required to be competent leaders, many programs target only primary chief residents [12].

**Figure 4:**
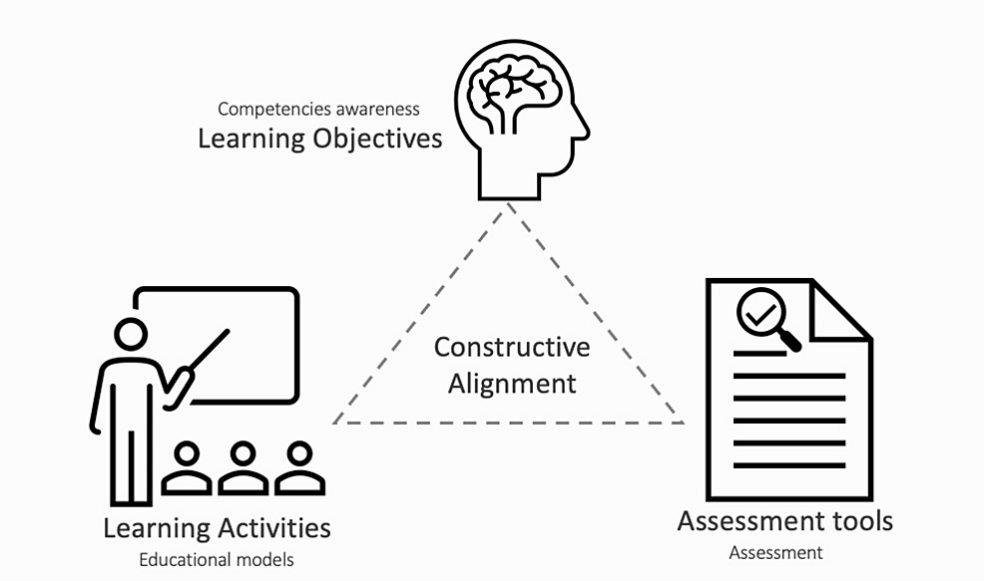
The role of constructive alignment in education; where the learning activities and assessments support students in achieving the learning objectives and outcomes

Second, although the leader role tackled as a competency required, our findings showed improper articulation between the key competencies' awareness as learning outcomes, educational models as activities, and assessment tools. Residents were highly motivated toward developing leadership as they believed it would help them succeed and achieve their goals. Nothing was relative to the outcome needed for their learning experience. Residents did not recognize specific educational models or assessment tools of leadership as part of a structured residency training program. That is contradictory to the role of constructive alignment in education, where the learning activities and assessments support students in achieving the learning outcomes (Figure 4) [13]. Separating the learning from the assessment would be counterproductive as the two are intimately linked [14]. All mentioned educational models and assessments were varied relative to the training centers and specialties. This discrepancy is likely because these activities and assessments were integrated into the training without assigning them to a specific curriculum component. Despite there being more teaching and assessment tools that have been recommended by CanMEDS, gaps existed in implementing the recommended ways to teach and assess leadership skills and behaviors. This affected the overall value of leadership and leadership development for the residents. Nevertheless, assessment tools of leadership behaviors during educational activities would allow reinforcement of the principles being taught [12]. Curriculum dissemination to all stakeholders is an important but often neglected step in curriculum implementation [15]. Therefore, attending physicians must be brought into the field so they become familiar with the basic leadership principles to the point where they can assess residents in real-life situations where these principles apply [11,12].

Third, residents place a high value on the learning environment [16]. They highly acknowledged how supervisors provide coaching and consider their feedback in addition to their scientific expertise. However, the residents showed their struggle and variations in developing leadership skills relying on their learning environment only. A recent study among internal medicine residents showed the same struggle that might provide inconsistent leadership background, which does not ensure equivalent development of leadership skills [17]. To support the development of leadership skills, leadership skills must become a standard part of medical training at all levels and in all specialties [5]. In addition, dovetail coaching and residents’ involvement in the program according to their needs [15,16].

Limitations

Limited generalizability was due to the inherent generalizability limitation in qualitative studies. This study was limited to a single dental residency training program institute, i.e., PSMMC. Thus, we are ignorant about other institutes. In addition, lack of peer-reviewed studies addressing leadership in residency in Saudi Arabia.

Strength

Our study addressed an important role in residency training related to the leader role as one of the competencies of the CanMEDS framework. This will open the gate for further studies and initiatives to improve leadership development in residency training programs.

## Conclusions

Our future leaders need to know the way, go the way, to show the way. This study explored leadership development and integrated training in the Saudi board dental residency program. It showed improper articulation between the learning outcomes, activities, and assessment. Residents struggled and varied in developing leadership skills relying on their educational experience and learning environment. Residency programs may verify constructive alignment and equivalent education of the “leader role” for all specialties and training centers in residency training in Saudi Arabia. Implement faculty development initiatives for appropriate learning and assessment of leadership skills. To our best knowledge, this is the first study about leadership education in residency training in Saudi Arabia. There is a need for a randomized multicenter study to measure competency level and engagement.
